# Detecting retinoblastoma

**Published:** 2018-06-03

**Authors:** Manoj V Parulekar

**Affiliations:** 1 Consultant Ophthalmologist: Birmingham Children's Hospital, Birmingham, London, UK.

**It is important to learn the early signs of retinoblastoma. Early detection, diagnosis and treatment depends on it.**

The most common early signs (see Table 1) of retinoblastoma are:

- Something white in the eye, often first noticed by parents. Confirm by conducting a red reflex test (see p. 23)
- Squint: one eye turns in or out (not as common).

Signs of more advanced retinoblastoma include:

- Forward displacement of the eye (proptosis)
- A visible tumour (fungating mass) involving just the globe, or extending to the orbit and/or the face.

Less common signs of retinoblastoma include:

- Poor vision, whether noticed by the parents or a health worker
- Nystagmus (constant eye movements)
- Hyphaema (bleeding into the anterior chamber)
- Pseudohypopyon (cells in the anterior chamber appearing as a layered white material)
- Periocular inflammation
- Phthisis bulbi (shrinkage of the globe)
- Raised pressure in the eye (glaucoma)
- Enlargement of the globe
- Change in iris colour (heterochromia).

## Retinoblastoma affecting both eyes

Signs of **germline retinoblastoma** are usually present in both eyes within the first few months of life (or even at birth in some instances). They include:

- Carers saying the eyes are not normal
- A white reflex in the pupil (or something white in the eye, noticed by the parents in one or both eyes)
- Occasionally nystagmus (searching eye movements as a result of the tumour involving the central part of the retina (macula) in both eyes).

## Retinoblastoma affecting one eye

Children with **non-germline retinoblastoma** usually present later, often at the age of 2–3 years. The most common presenting features in patients with non-germline retinoblastoma are **leucocoria** (an abnormal white reflection from the retina) and **squint** (less common).

**Table 1:** Could this be retinoblastoma?

	What the parents say, or what you can see	Could this be retinoblastoma?
**Listen to the child's carer**	The parent/carer says they saw something white in the eye	Yes
**Look at the child's eyes**	The eyes look normal but the parent/carer says they saw something white in the eye	Yes
	The eyes are not looking in the same direction; they are not straight	Squint is quite common in children It is rarely due to retinoblastoma
	One of the pupils is not black; it may be white or yellow-orange	This could be cataract or retinoblastoma
	One of the eyes is bigger than the other or bulges forward	This may be proptosis due to advanced retinoblastoma
**Examine the pupil and red reflex**	Look at the pupils. They should be black. Do a red reflex test (p. 23)	Suspect retinoblastoma if anything in the pupil looks abnormal

**Figure:**
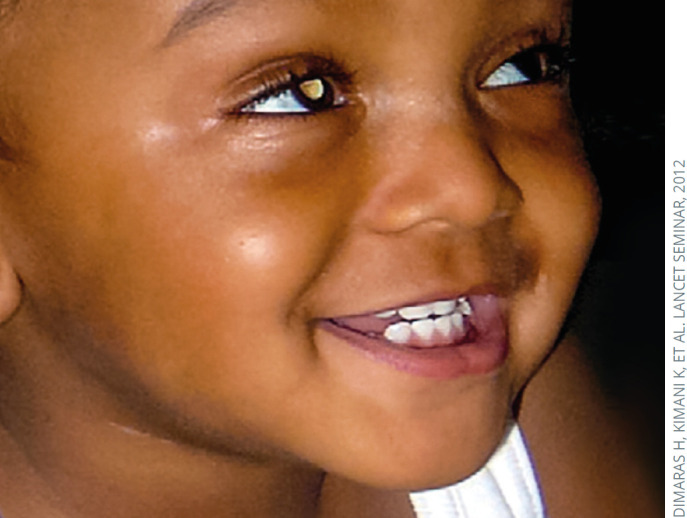
Leucocoria. An abnormal or white reflex is the most common sign of retinoblastoma.

**Note:** If a child presents with unilateral retinoblastoma in the first year of life, it may be germline (p. 7). The child has a greater risk of developing more tumours in the same or the other eye. Careful screening of the fellow eye, throughout childhood, is essential for all children suspected of having germline disease.

## What are you likely to see where?

In high-income countries, children with retinoblastoma may present with reduced vision, an acute red eye or orbital inflammation.

In low resource settings advanced cases with proptosis (forward displacement of the eye) or fungating orbital masses are seen more commonly due to late presentation.

**Figure:**
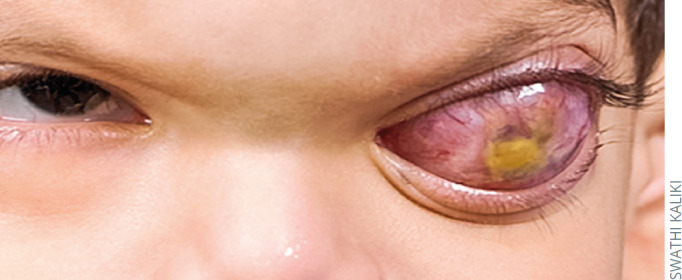
More advanced retinoblastoma. INDIA

**Digital flash photography**

Digital flash photography, often from a mobile phone, can show the white reflex in the pupil in some cases and lead to earlier detection; however, squint, refractive errors, and photographs taken from an angle can also produce the appearance of a white reflex, with only a minority of cases having retinoblastoma.

So, although most causes of an absent red pupil reflex on a photograph are not retinoblastoma it is still a useful public education tool. In Honduras, it was associated with a reduction in the proportion of children with retinoblastoma presenting with advanced disease.

